# New developments in the molecular treatment of ichthyosis: review of the literature

**DOI:** 10.1186/s13023-022-02430-6

**Published:** 2022-07-15

**Authors:** M. D. W. Joosten, J. M. K. Clabbers, N. Jonca, J. Mazereeuw-Hautier, A. H. Gostyński

**Affiliations:** 1 Department of Dermatology, The Netherlands and European Reference Network – Skin, Maastricht University Medical Center, Maastricht, The Netherlands; 2 GROW School for Oncology and Developmental Biology, Maastricht University Medical Center, P. Debyelaan 25, 6229HX Maastricht, The Netherlands; 3 Department of Dermatology, Haga Hospital, The Hague, The Netherlands; 4 Cell Biology and Cytology Laboratory, CNRS, Inserm, UPS, European Reference Network – Skin, University Hospital Center of Toulouse and Infinity, Federal Biology Institute, Toulouse University, Toulouse, France; 5 Department of Dermatology, European Reference Network – Skin, University Hospital Center of Toulouse, Toulouse, France

**Keywords:** Ichthyosis, Management, Gene therapy, Replacement therapy, Biological therapy, Small molecule therapy

## Abstract

Ichthyosis covers a wide spectrum of diseases affecting the cornification of the skin. In recent years, new advances in understanding the pathophysiology of ichthyosis have been made. This knowledge, combined with constant development of pathogenesis-based therapies, such as protein replacement therapy and gene therapy, are rather promising for patients with inherited skin diseases. Several ongoing trials are investigating the potency of these new approaches and various studies have already been published. Furthermore, a lot of case series report that biological therapeutics are effective treatment options, mainly for Netherton syndrome and autosomal recessive congenital ichthyosis. It is expected that some of these new therapies will prove their efficacy and will be incorporated in the treatment of ichthyosis.

## Background

**Fig. 1:**
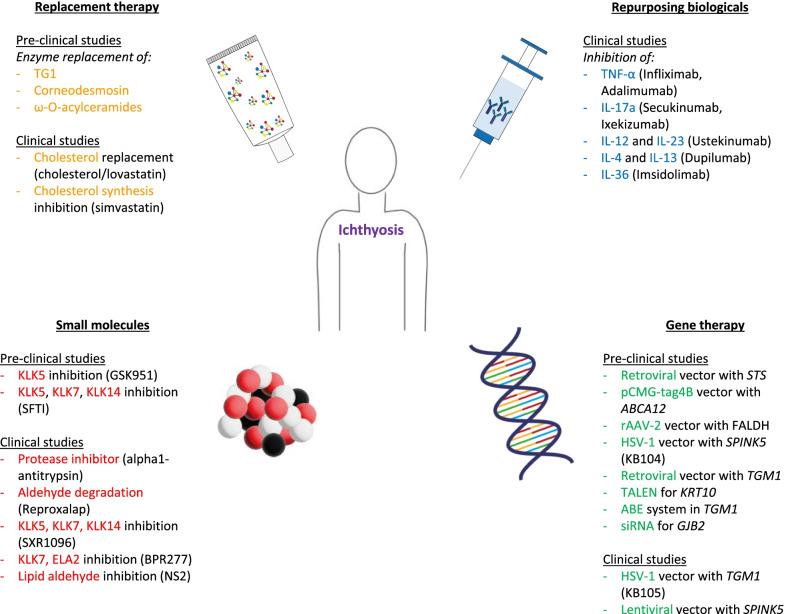
Overview of the recent developments in molecular treatment of ichthyosis. *ABE* adenine base editor, *ELA2* epidermal elastase 2, *HSV-1* herpes-simplex virus-1, *IL* interleukin, *KLK* kallikrein-related peptidase, *rAAV-2* recombinant adeno-associated virus-2, *siRNA* small interfering RNA, *TALEN* transcription activator-like effector nuclease, *TG1* transglutaminase-1, *TNF-α* tumor necrosis factor alpha

Ichthyosis is a group of heterogeneous disorders affecting the cornification of the skin. Many ichthyosis variants are inherited and a subdivision is made between syndromic and non-syndromic variants (Table 1) [1]. All forms of ichthyosis are characterized by extensive scaling, hyperkeratosis, and often inflammation of the skin, resulting in erythroderma. Many patients report anxiety and depression and most of them experience a quality of life impairment [2–4]. Current treatment for ichthyosis is focused on symptom relief and includes emollients, keratolytics, and oral retinoids. The efficacy of these treatments is moderate and is usually not effective on inflammation of the skin [1, 5–7]. In the past few years, new advances in understanding the pathophysiology of ichthyosis have been made [8, 9]. Promising developments have been made in pathogenesis-based therapies, such as enzyme replacement therapy and gene therapy, and recent findings concerning the immune profile of ichthyosis patients have given new ground to repurpose biologicals (Fig. 1). The aim of this review is to provide an overview of the current status on pathogenesis-based therapy for ichthyosis. A Pubmed search was performed with the terms ichthyosis, therapeutics, biological products, molecular targeted therapy, enzyme replacement therapy and genetic therapy. Relevant articles were selected based on title and abstract. Selected articles are summarized in Table 2. Information about current ongoing clinical trials was retrieved from www.clinicaltrials.gov and summarized in Table 3.

**Table 1:** Brief overview of common ichthyoses and the underlying gene mutations

Name	Gene mutation
*Non-syndromic*	
*Common*	
Ichthyosis vulgaris	*FLG*
X-linked recessive ichthyosis	*STS*
* Autosomal recessive congenital ichthyosis*	
Lamellar ichthyosis—Congenital ichthyosiform erythroderma spectrum	*ABCA12, ALOXE3, ALOX12B, CASP14, CERS3, CYP4F22, LIPN, NIPAL4, PNPLA1, SDR9C7, ST14, SUBLT2B1, TGM1*
Harlequin ichthyosis	*ABCA12*
Bathing suit ichthyosis	*TGM1*
* Keratinopathic ichthyoses*	
Epidermolytic ichthyosis	*KRT1, KRT10*
Superficial epidermolytic ichthyosis	*KRT2*
Congenital reticular ichthyosiform erythroderma	*KRT1, KRT10*
*Other*	
Peeling skin syndrome type 1	*CDSN*
*Syndromic*	
Netherton syndrome	*SPINK5*
Sjögren–Larsson syndrome	*ALDH3A2*
Severe skin dermatitis, multiple allergies and metabolic wasting syndrome	*DSG1, DSP*
Keratitis-ichthyosis-deafness syndrome	*GJB2, AP1B1*

Modified after Oji V et al. J Am Acad Dermatol. 2010 and Fischer J, Bourrat E., Acta Derma Venereol. 2020 [1, 10]

**Table 2:** Overview of recently published pathogenesis-based therapies for ichthyosis, surveyed in text

Year	Study	Article	Disease	Treatment
*Preclinical studies*				
2021	Liddle et al*.* [30]	Original research	NS	Small molecule; KLK5 inhibitor (GSK951)
2019	Chen et al*.* [77]	Original research	NS	Small molecule; Sunflower Trypsin Inhibitor (SFTI)
2013	Aufenvenne et al*.* [82]	Original research	ARCI (*TGM1*)	Replacement therapy; TG1
2019	Plank et al*.* [84]	Original research	ARCI (*TGM1*)	Replacement therapy; TG1
2021	Valentin et al*.* [36]	Original research	PSS1	Replacement therapy; corneodesmosin
2017	Grond et al*.* [85]	Original research	ARCI (*PNPLA1*)	Replacement therapy; PNPLA1
2018	Mauldin et al*.* [86]	Original research	ARCI (*NIPAL4*)	Replacement therapy; ω-O-acylceramides
1997	Freiberg et al*.* [99]	Original research	XLI	Gene therapy; *STS* in retroviral vector
2005	Akayima et al*.* [20]	Original research	HI	Gene therapy; corrective gene transfer
2005, 2006	Haug et al*.* [33, 100]	Original research	SLS	Gene therapy; FALDH in rAAV-2
2019	Bustos et al	Original research (abstract)	NS	Gene therapy; *SPINK5* in HSV-1
1996	Choate et al*.* [102]	Original research	LI	Gene therapy; *TGM1* in a retroviral vector
2021	Freedman et al*.* [103]	Original research	ARCI (*TGM1*)	Gene therapy; *TGM1* in HSV-1
2019	March et al*.* [101]	Original research	EI (*KRT10*)	Gene therapy; TALEN
2022	Dang et al. [105]	Original research	LI	Gene therapy: base editing
2020	Lee et al*.* [106]	Original research	KID	Gene therapy; siRNA
*Clinical studies*				
2011	Fontao et al*.* [43]	Case report	NS	Biological therapeutic; infliximab
2017	Roda et al*.* [44]	Case report	NS	Biological therapeutic; infliximab
2020	Steuer et al*.* [60]	Case report	NS	Biological therapeutic; dupilumab
2020	Andreasen et al*.* [61]	Case report	NS	Biological therapeutic; dupilumab
2021	Wang et al*.* [62]	Case report	NS	Biological therapeutic; dupilumab
2021	Sussmuth et al*.* [63]	Case series	NS	Biological therapeutic; dupilumab
2021	Murase et al*.* [64]	Case series	NS	Biological therapeutic; dupilumab
2020	Aktas et al*.* [65]	Case report	NS	Biological therapeutic; dupilumab
2020	Volc et al*.* [56]	Case report	NS	Biological therapeutic; ustekinumab
2020	Luchsinger et al*.* [51]	Case series	NS	Biological therapeutic; secukinumab
2020	Blanchard et al*.* [52]	Case report	NS	Biological therapeutic; secukinumab
2021	Barbieux et al*.* [54]	Case series	NS	Biological therapeutic; ixekizumab
2019	Poulton et al*.* [66]	Case report	ARCI (*NIPAL4*)	Biological therapeutic; ustekinumab
2019	Haiges et al*.* [67]	Case report	CIE, not genetically proven	Biological therapeutic; secukinumab
2019	Hernandez-Martin et al*.* [68]	Case report	SAM syndrome	Biological therapeutic; secukinumab
2018	Paller et al*.* [69]	Case series	SAM syndrome	Biological therapeutic; ustekinumab
2022	Lefferdink et al*.* [70]	Randomized controlled trial	EI NS LI CIE	Biological therapeutic; secukinumab
2006	Mazereeuw-Hautier et al*.* [78]	Randomized controlled trial	NS	Small molecule; rAAT
2011	Pallet et al*.* [87]	Case series	CHILD syndrome	Cholesterol replacement; 2% cholesterol and 2% lovastatin
2018	Bergqvist et al*.* [42]	Case series	CHILD syndrome	Cholesterol replacement; 2% cholesterol and 2% lovastatin
2019	Sandoval et al*.* [88]	Case report	CHILD syndrome	Cholesterol replacement; 2% cholesterol and 2% lovastatin
2019	Yu et al*.* [89]	Case series	CHILD syndrome	Cholesterol replacement; 2.5% and 5% simvastatin
2022	Kallis et al*.* [90]	Case report	CHILD syndrome	Cholesterol replacement; 5% simvastatin
2018	Bajawi et al*.* [91]	Case report	CHILD syndrome	Cholesterol replacement; 2% simvastatin
2019	Di et al*.* [107]	Clinical trial	NS	Gene therapy; lentiviral vector *(SPINK5);* epidermal sheet generation

*ARCI* autosomal recessive congenital ichthyosis, *CIE* congenital ichthyosiform erythroderma, *CHILD* syndrome congenital hemidysplasia with ichthyosiform erythroderma and limb defects, *EI* epidermolytic ichthyosis, *HI* Harlequin ichthyosis, *HSV* herpes simplex virus, *KID* keratitis–ichthyosis–deafness syndrome, *KLK5* kallikrein 5, *LI* lamellar ichthyosis, *NS* Netherton syndrome, *PSS1* peeling skin syndrome type 1, *rAAT* recombinant alpha antitrypsin, *rAAV* adeno-associated virus-2 vectors, *SAM* *syndrome* severe dermatitis, multiple allergies and metabolic waisting syndrome, *siRNA* small interfering RNA, *SLS* Sjögren–Larsson syndrome, *STS* steroid sulfatase, *TALEN* transcription activator-like effector nuclease, *TGM1* transglutaminase, *XLI* X-linked recessive ichthyosis

**Table 3:** Current and recently completed registered clinical trials, awaiting for results to be published

Phase (ID)	Condition	Type of treatment	Approach	Participants (N)	Status	Reference
I (NCT04549792)	Ichthyosis, not specified	Biological therapeutic	Antibody targeting IL-12/IL-23; Ustekinumab	15	Ongoing	https://clinicaltrials.gov/ct2/show/NCT04549792
II (NCT02113904)	NS	Biological therapeutic	Antibody targeting TNF-α; Adalimumab	11	Completed	https://clinicaltrials.gov/ct2/show/NCT02113904
II/III (NCT04244006)	NS	Biological therapeutic	Antibody targeting IL-4 and IL13; Dupilumab	24	Ongoing	https://clinicaltrials.gov/ct2/show/NCT04244006
II (NCT04697056)	Ichthyosis, not specified	Biological therapeutic	Antibody targeting IL-36 receptor; Imsidolimab	24	Ongoing	https://clinicaltrials.gov/ct2/show/NCT04697056?cond=ichthyosis&draw=2&rank=3
IV (NCT04996485)	Ichthyosis, children	Biological therapeutic	Antibodies targeting IL-17 (Secukinumab), IL-12/IL-23 (Ustekinumab), IL-4/IL-13 (Dupilumab)	50	Ongoing	https://clinicaltrials.gov/ct2/show/NCT04996485?cond=ichthyosis&draw=3&rank=6
I (NCT01428297)	NS	Small molecule	KLK7 and ELA2 inhibitor; BPR 277	12	Completed	https://clinicaltrials.gov/ct2/show/NCT01428297?cond=netherton&draw=2&rank=7
III (NCT03445650)	SLS	Small molecule	Reactive Aldehyde Species (RASP) inhibitor; Reproxalap	11	Completed	https://clinicaltrials.gov/ct2/show/NCT03445650
II (NCT02402309)	SLS	Small molecule	Aldehyde binding small molecule; NS2 cream	12	Completed	https://clinicaltrials.gov/ct2/show/NCT02402309?cond=ichthyosis&draw=3&rank=23
I/II (NCT05211830)	NS	Small molecule	Topical application of a new developed protease inhibitor (SXR1096)	20	Ongoing	https://clinicaltrials.gov/ct2/show/NCT05211830
I/II (NCT04047732)	ARCI (*TGM1*)	Gene therapy	Non-integrating HSV-1 vector expressing TGM1 as a topical gel (KB105)	6	Ongoing	https://clinicaltrials.gov/ct2/show/NCT04047732
I (NCT01545323)	NS	Gene therapy	Grafting autologous epidermal sheets from genetically modified skin stem cells	5	Unknown	https://clinicaltrials.gov/ct2/show/NCT01545323

*TGM1* transglutaminase-1, *ARCI* autosomal recessive congenital ichthyosis, *LI* lamellar ichthyosis, *CIE* congenital ichthyosiform erythroderma, *EI* epidermolytic ichthyosis, *ELA2* epidermal elastase 2, *KLK7* kallikrein-related peptidase 7, *NS* Netherton syndrome, *SLS* Sjögren–Larsson syndrome, *IL* interleukin, *HSV-1* herpes simplex virus-1, *TNF- α* tumor necrosis factor alpha

## Molecular pathology of ichthyosis

Mutations in over 50 genes are known to cause an ichthyosis phenotype (Table 1)*.* [10] Four processes in skin cornification can be involved in the pathophysiology, i.e., the process of desquamation, impairment in the keratin synthesis, impairment in the synthesis of the cornified envelope or impairment in the organization of the stratum corneum extracellular lipid matrix. This leads to an altered epidermal differentiation, a defective epidermal barrier and increased transepidermal water loss (TEWL) [1, 5–7]. Below we will focus on the pathophysiology of the ichthyosis subtypes for which pathogenesis-based treatments have emerged in the past years.

X-linked recessive ichthyosis (XLI) is caused by mutations in the steroid sulfatase (*STS*) gene and is usually present soon after birth. It is characterized by hyperkeratosis and generalized polygonal brown scales. Extracutaneous associations include protracted delivery and cryptorchidism. Steroid sulfatase is responsible for the breakdown of cholesterol sulfate, necessary for corneodesmosome degradation and normal desquamation. A steroid sulfatase deficiency leads to accumulation of cholesterol sulfate in the stratum corneum resulting in impaired desquamation [7, 11].

Autosomal recessive congenital ichthyosis (ARCI) describes a spectrum of ichthyoses including lamellar ichthyosis (LI), congenital ichthyosiform erythroderma (CIE), and Harlequin ichthyosis (HI). The phenotype of each subtype differs. Children are often born with a collodion membrane. LI is characterized by rough, dark brown scaling, palmoplantar keratoderma and scarring alopecia. CIE expresses as erythroderma with fine white scaling. HI presents itself at birth with a very thick and rigid collodion membrane causing ectropion, eclabium, restriction of movements, and high risk of mortality. The genetic defect in ARCI is based on mutations in numerous genes (Table 1), [1, 7, 12, 13]. The most common form of ARCI is caused by mutations in the transglutaminase-1 (*TGM1*) gene, which encodes for the transglutaminase-1 (TG1) protein [10]. TG1 mediates crosslinking of cytoplasmic proteins onto the plasma membrane to form the cornified envelope. The lipoxygenase-hepoxilin and acylceramide pathways are essential for formation of the corneocyte lipid envelope and extracellular lipid membranes. Mutations in genes involved in these pathways account for most other forms of ARCI. The *NIPAL4* and *PNPLA1* genes will be elaborated more in detail, as these genes are involved in recent developments regarding replacement therapy [12, 14, 15]. *NIPAL4* encodes for ichthyin, a transmembrane protein in the granular layer of the epidermis. It is thought to be a Mg^2+^ -transporter that contributes to lipid metabolism in the epidermal development. When the ichthyin function is defective, there will be decreased levels of acylceramide and impaired lipid structures of the stratum corneum, that may be related to the skin permeability barrier defect [16, 17]. *PNPLA1* plays a role in the synthesis of ω-O-acylceramide. This lipid is a key component for the permeability layer of the epidermis. In the stratum corneum of *PNPLA1*-deficient humans, ω-O-acylceramide loss is linked to impairment of the corneocyte lipid envelope and the extracellular lipid lamellae [18]. HI is caused by mutations in the *ABCA12* gene. ABCA12 is an epidermal keratinocyte lipid transporter, involved in secretion of lipids, and is mainly localized in the granular layer. Mutations in *ABCA12* result in a loss of the lipid skin barrier [19, 20].

Epidermolytic ichthyosis (EI) is a form of ichthyosis that is characterized by congenital erythroderma, hyperkeratosis and blistering. EI is caused by autosomal dominant mutations in the keratin-1 (*KRT1)* or the keratin-10 (*KRT10)* gene, encoding for keratin 1 and keratin 10 proteins, respectively [1]. Mutations in the *KRT1* and *KRT10* genes induce clumping of the keratin intermediate filament (KIF) network in suprabasal keratinocytes, and cellular collapse. The mutations may also interfere with lamellar body secretion and therefore the lipid membrane formation, causing impaired barrier function [12, 21–23].

Congenital reticular ichthyosiform erythroderma (CRIE) results in a reticular ichthyosiform phenotype with yellow–brown scaling and erythroderma. It is due to autosomal dominant mutations in the tail regions of the *KRT1* and the *KRT10* gene, also causing collapse of the KIF network [24, 25]. Affected patients develop multiple confetti-like spots, that are genotypically wild-type and increase in surface and amount with growing age. This phenomenon is called revertant mosaicism and is caused mostly by a mechanism called mitotic recombination that results in somatic homozygosity of the wildtype allele [1, 26, 27].

Netherton syndrome (NS) is characterized by congenital scaly erythroderma, evolving into typical erythematous patches with peripheral scaling (ichthyosis linearis circumflexa), hair shaft abnormalities (trichorrhexis invaginata/bamboo hair) and atopic manifestations. It is an autosomal recessive disorder caused by mutations in the *SPINK5* gene [1]. This gene encodes for lympho‐epithelial Kazal‐type‐related inhibitor (LEKTI). LEKTI is a serine protease inhibitor and regulates the degradation of corneodesmosomes by kallikrein-related peptidases (KLKs). KLK5 and matriptase cause a cascade that activates other KLKs, like KLK7 and KLK14, and elastase 2. In NS, there is a LEKTI deficiency resulting in unrestrained KLK activation in the epidermis. This obstructs stratum corneum cohesion and therefore leads to detachment of the stratum corneum, causing a severe permeability barrier defect. Furthermore, KLK5 and KLK14 activation results in the activation of proteinase-activated receptor 2 (PAR-2), which leads to synthesis of pro-inflammatory factors in keratinocytes [1, 28–30].

Sjögren-Larsson syndrome (SLS) is an autosomal recessive disorder involving the skin, eyes and central nervous system. The *ALDH3A2* gene encodes for fatty aldehyde dehydrogenase (FALDH). This enzyme catalyzes the oxidation of fatty aldehyde from various lipid pathways. Mutations in the *ALDH3A2* gene, lead to a FALDH deficiency. The fatty alcohols will accumulate and are diverted to other lipids, which may interfere with normal formation of lamellar body membranes in keratinocytes and lead to abnormal stratum corneum membrane. The accumulated fatty alcohols are thought to interfere with the function of the myelin membranes in the central nervous system, leading to neurological symptoms [1, 31–33].

SAM syndrome stands for severe dermatitis, multiple allergies and metabolic wasting syndrome. This congenital form of ichthyosis is due to mutations in the desmoglein-1 (*DSG1*) or the desmoplakin (*DSP*) gene, encoding for desmoglein-1 and desmoplakin, respectively. Both proteins are crucial components of desmosomes, necessary to connect the cell surface to the KIF cytoskeleton. Mutations in the *DSG1* or *DSP* gene cause loss of cell-to-cell adhesion and differentiation disturbances [34, 35].

Peeling skin syndrome type 1 (PSS1) has clinically some resemblances with NS. PSS1 is characterized by erythroderma and superficial peeling of the skin. It is caused by mutations in the corneodesmosin gene (*CDSN*)*.* Lack of corneodesmosin results in subcorneal splitting and detachment of corneocytes [36].

Keratitis-ichthyosis-deafness (KID) syndrome comprises a vascularizing keratitis, erythrokeratoderma skin lesions and a sensorineural hearing loss. KID syndrome is caused by mutations in the connexin-26 (Cx26) gene *(GJB2).* Gap junctions regulate cellular communication and activities, and are composed of multiple connexins. Cx26 is one of these connexins and is expressed in many epithelial organs, including the inner ear and the skin. Mutations in the *GJB2* gene cause a dysfunction of Cx26 and therefore a dysfunction of the gap junctions [37–40].

CHILD syndrome (congenital hemidysplasia with ichthyosiform erythroderma and limb defects) is a cutaneous mosaicism, caused by mutations in the NAD(P)H steroid dehydrogenase-like (*NSDHL*) gene, which is involved in the process of cholesterol synthesis. The mutation leads to deficiency of bulk cholesterol, which is thought to adjust the keratinocyte membrane and the skin barrier, resulting in accumulation of upstream potentially toxic metabolic intermediates [41, 42].

## Overview of new therapies in development for ichthyosis

### pathways

Biological therapy includes a wide range of products, such as monoclonal antibodies that aim on targeting specific marks, e.g., tumor necrosis factor alpha (TNF-α), interleukin-13 (IL-13), interleukin-17 (IL-17) and interleukin-23 (IL-23). There is substantial experience in the usage of biologics in inflammatory skin diseases, such as psoriasis and atopic dermatitis (AD). Since they are already commercially available and dermatologists are experienced in their usage, the path for clinical testing could be rather rapid [9].

In 2011, Fontao et al. [43] provided the first report of a patient with NS who was successfully treated with infliximab, an inhibitor of TNF-α. After 12 weeks of treatment, the skin was clear of inflammatory lesions, however xerosis and ichthyosis did not improve. In 2017, Roda et al. [44] reported another NS patient who experienced a clearance of inflammatory lesions after initiation of infliximab. Nonetheless, TNF-α inhibitors are not recommended in the treatment of NS according to the European guideline of care for ichthyosis, due to paucity of data, risk of non-melanoma skin cancers and recurrent infections [45].

Only recently, the repurposing of other biologics has been proposed. The rationale of repurposing biologics for ichthyosis is based on studies of Paller et al*.* [46–48] In one of their studies (2017) [46], the immune profile of LI, EI, CIE and NS was examined and compared to healthy controls as well as AD and psoriasis patients. An IL-17 dominant profile, similar to psoriasis, was found in all forms of ichthyosis. NS patients showed the highest induction of IL-17 and interleukin-22 (IL-22) pathway genes. The IASI-E (Ichthyosis Area and Severity index—erythema) score showed a significant correlation with IL-17A levels and TEWL. Similar results were shown in the studies that followed, where elevated IL-17 and TNF-α levels in skin and blood samples were found [47, 48]. An important but still unanswered question is whether the cutaneous symptoms in ichthyosis are caused by the overactive Th17 pathway, or if Th17 activation is only a sign of a systemic immune response without a pathogenic relationship to the skin abnormalities [49].

Secukinumab is a recombinant human monoclonal antibody that targets the IL-17A cytokine [50]. Luchsinger et al*.* [51] reported a case series of four patients with NS that were treated with secukinumab during 3–12 months. Overall, they reported a reduction in IASI from 55 to 88% after 6 months. However, response was less pronounced in two patients with a milder variant of NS. Blanchard et al*.* [52] reported a case of a patient with NS who had facial erythema and frequent flares of ichthyosis on the trunk and extremities. Treatment with secukinumab resulted in complete clearance of facial erythema, with one mild flare of plaques in three years of follow-up.

Another biological therapeutic that targets IL-17A is ixekizumab, which seems to have higher affinity for IL-17A than secukinumab [53]. Barbieux et al*.* [54] reported three NS patients, two with the ichthyosis linearis circumflexa (NS-ILC) phenotype and one patient with a scaly erythroderma (NS–SE) phenotype. They all had enhanced levels of IL-17 related cytokines and were treated with ixekizumab for 6 months. IASI-S, Dermatology Life Quality Index (DLQI) and pruritus scores showed a significant reduction of about 50% after 3 months. The IASI-E scores were only reduced for the NS-ILC patients. After 6 months, all scores remained decreased in the NS-ILC patients. Regarding the NS-SE patient, all scores returned back to baseline, except for the IASI-E score.

Ustekinumab indirectly inhibits IL-17 by targeting interleukin-12 (IL-12) and interleukin-23 (IL-23) [55]. Volc et al*.* [56] reported a 15-year-old girl with NS who was treated with ustekinumab. The skin improved substantially within 4 weeks and no relapse occurred after one year. Ustekinumab has also been used in *CARD14* associated papulosquamous eruption (CAPE), an inflammatory dermatosis with palmoplantar hyperkeratosis and erythroderma. The familial variant is caused by mutations in the *CARD14* gene, which is known to be an activator of the noncanonical nuclear factor-kappa B (NF-κB) pathway, which leads to inflammation. Affected patients express an increase in CARD14 in the epidermis. Case reports show benificial effects for CAPE patients who have been treated with ustekinumab [57–59].

Dupilumab is used in the treatment of AD. It blocks the interleukin-4 receptor and therefore inhibits IL-4 and interleukin-13 (IL-13). IL-4 and IL-13 stimulate the Th2 cells, that regulate the pro-allergic adaptive immune response. Dupilumab has been proposed for NS, since the clinical presentations of NS and eczema show resemblances. A few case reports, that report a total of 5 NS patients, describe that dupilumab is effective in NS [60–63]. Süssmuth et al*.* [63] described a 12-year-old girl and an 8-year-old boy with NS that were treated with dupilumab. After 4 months, the Netherton Area Severity Assessment (0–72) dropped from 33 to 11.7 for the girl and from 50.5 to 18 for the boy. For both children, the pruritus dropped from 8 to 3 on the numeric rating scale (NRS; 0–10). These results remained stable after 12 months of treatment. However, Murase et al*.* [64] reported two cases of NS patients who were treated with dupilumab, where the pruritus gradually reoccurred in the second week after the dupilumab injection. In a case report from Aktas et al*.* [65] the skin lesions and itch worsened after an initial good response during the first 6 weeks of therapy.

A limited number of case reports describe usage of biological therapy in the other subtypes of ichthyosis. One case reports a 4-year-old boy with ARCI, based on a *NIPAL4* mutation, and a large joint inflammatory arthropathy of unknown origin. He was given ustekinumab, which gave a good response at first, but after 12 weeks a relapse occurred. A dosage increase resulted in a clearance of erythema and no further joint complaints [66]. Haiges et al*.* [67] reported a 20-year-old man with a clinical diagnosis of CIE, but genetic testing did not disclose a mutation in ichthyosis associated genes. Treatment with secukinumab was started based on a biopsy showing an inflammatory infiltrate rich in IL-17. Significant improvements in scaling and erythroderma were reached, however punch biopsies showed that acanthosis, hyper- and parakeratosis were only slightly reduced.

Hernández-Martin et al*.* [68] presented a case about a 9-month-old girl with a clinical presentation of SAM syndrome, including erythroderma, hyperkeratosis, pruritus, recurrent episodes of sepsis and severe growth retardation. The genetic analysis did not show a mutation in the *DSG1* gene. However, the immunofluorescence staining showed abnormalities in the desmosomes. The patients’ IL-17-producing T-cell frequency was 67 times higher compared to healthy controls. She was given 75 mg of secukinumab every week for the first 4 weeks, which was then continued on a monthly basis. At week 35 of treatment, the pruritus had almost disappeared and the weight-for-height z score had increased from − 2.3 SD to + 1.9 SD. Th17 and IL-17 producing T cell levels were more than halved. Another case report described two patients with SAM syndrome due to mutations in the desmoplakin gene. Treatment with ustekinumab resulted in a 58% and 59% reduction in total IASI and a lower TEWL by 16 weeks [69].

Results from a double-blind placebo-controlled study with secukinumab in different subtypes of ichthyosis from Lefferdink et al*.* [70] have recently been published. Included patients had a diagnosis of EI, NS, CIE or LI and received secukinumab or placebo every 4 weeks for 16 weeks in total, followed by a 16-week open-label phase and a 20-week extension for safety. IASI, the Visual Index for Ichthyosis Severity (VIIS), TEWL, and patient reported outcomes were measured. Furthermore, skin biopsies were taken to study biomarkers of the Th17 pathway. There was no significant difference between secukinumab and placebo group in ichthyosis severity scores, TEWL and patient reported outcome measures at week 16. The group who received secukinumab first, only had a significant reduction in IASI-E and VIIS at week 32 and in VIIS at week 52. A total of five patients (two with NS; two with EI; one with CIE) continued the treatment post-study because of self-perceived improvement. These patients had a median total IASI decrease of 36% (range 29–50%) at the end of the study. Th17 related biomarkers did not show a significant reduction in week 16 and 32 compared to baseline. There was a lack of response in all LI patients, which suggest that only a few ichthyosis subtypes respond to IL-17 treatment. The study was performed on a limited population with different subtypes, resulting in small subgroups. Unfortunately authors did not provide information about genetic mutations of the treated subjects so further analysis per subtype is not possible.

In conclusion, the use of biological therapies could be beneficial in the treatment of several ichthyosis subtypes. However, the response is mostly seen as reduction of the inflammatory component and pruritus. The effect on scaling seems to be more variable. A larger randomized controlled trial (RCT) and/or large open-label cohort is necessary to rule out a possible publication bias and result in more specific data and correlation with genetic mutations and immunological profile. This could help to predict which subtypes, phenotypically and genetically, show better response to anti IL-17 therapy.

Furthermore, there are case reports that describe a good response at first, but a decline after several weeks [54, 64–66]. Perhaps biological therapies in ichthyosis patients require an increased dosage and/or interval compared to the currently used regimen indicated for psoriasis and atopic dermatitis. Most case reports describe effects on young patients. It is possible that also the length of disease affects the therapeutic response. In addition, it would be interesting to further investigate if certain biomarkers, such as increased cytokine levels, could help to choose between the different biological therapies [49]. It is proposed that upstream molecules, such as IL-23 or IL-36 may have a better effect [70]. Several registered clinical trials are ongoing and could provide more information regarding the use of all these different biological therapies. These ongoing clinical trials (Fig. 1) include studies regarding dupilumab (anti IL-4), adalimumab (anti TNF-α), ustekinumab (anti IL-12/IL-23) and imsidolimab (anti IL-36).

### Small molecules

Small molecules are used to inhibit certain proteins, such as protein kinases. Because of their size and molecular properties, they are able to interact with specific parts of the targeted protein and therefore inhibit it without disrupting the pathways of other proteins [71]. They are currently used to treat inflammatory skin diseases, such as apremilast in psoriasis treatment and baricitinib for atopic dermatitis. For these indications, small molecules have shown promising results [72, 73]. Regarding ichthyosis, only a few in vitro and mouse-model studies have been conducted in this field.

#### Preclinical studies

Small molecules could be applicable in NS, where the dysfunctional *SPINK5* leads to an unopposed KLK5 activity. Several studies have reported that knockdown or ablation of KLK5 leads to improvements of NS in in vivo mouse-models and in in vitro skin models with human epidermal keratinocytes [74–76]. Liddle et al*.* [30] discovered GSK951, a small molecule inhibitor of KLK5. GSK951 was applied topically for three days to a transgenic murine model expressing human KLK5.After day one, the TEWL was decreased to approximately 50%. However, the mice that were treated topically with the vehicle cream without GSK951 had a two-fold increase of TEWL. Thus, it is not possible to state that the GSK951 causes the TEWL decrease. However, this data does show that topical application of GSK951 is able to inhibit KLK5 activity in the skin. Furthermore, the inflammatory cytokines did show a significant decrease, mainly the IL-17 cytokines.

Chen et al*.* [77] showed the potency of Sunflower Trypsin Inhibitor (SFTI) to treat NS. SFTI is a small peptide and potent inhibitor of trypsin. Analogues of SFTI can be used to redirect its inhibitory capacities to other proteases. In this study, they showed that the developed SFTI is able to effectively inhibit KLK5, KLK14 and KLK7. The inhibitory potency has not been tested in animal or human skin models so far.

#### Clinical studies

Mazereeuw-Hautier et al*.* [78] conducted an in vivo study with NS patients, where they used a protease inhibitor alpha1-antitrypsin, similar to the missing protease inhibitor (LEKTI) in NS. They performed a RCT using recombinant human alpha1-antitrypsin gel and placebo for 21 days. Erythema and scaling were scored, but results did not show statistically significant differences.

A new clinical trial has been initiated in 2021, investigating application of a cream with substance SXR1096 in NS patients during 1 month (NCT05211830). SXR1096 is a potent inhibitor of KLK5, KLK7 and KLK14. Twenty NS patients will participate in this phase I/II trial to assess the safety and effectiveness as compared to placebo [79].

In 2018, Aldeyra Therapeutics Inc [80]. announced their phase III randomized double-blind vehicle-controlled trial with topical application of ADX-102 1% (Reproxalap) in SLS patients (NCT03445650), which has now been completed. ADX-102 is a small molecule, able to degrade aldehydes, and could be helpful to reduce the lipid accumulation as result of the FALDH deficiency in SLS. Results of this trial are not available yet.

Besides the trials described above, two other registered clinical trials are still ongoing (Fig. 1), considering treatments for NS and SLS. These trials include the use of BPR 277, a KLK7 and epidermal elastase 2 (ELA2) small molecule inhibitor in NS, and NS2 cream, an aldehyde binding small molecule for SLS.

### Replacement therapy

Enzyme replacement therapy (ERT) entails the replacement of a deficient structural protein or enzyme. It has been used for over 20 years for lysosomal storage disorders like Gaucher and Fabry disease, where it has shown high efficacy [81]. For ichthyosis, it could be applicable for recessive forms, where a deficiency of a specific protein leads to the phenotype. For systemic diseases, as Fabry disease, systemic application is already available, while for ichthyosis only topical treatment has been tested so far [82]. Lipid replacement is also a promising approach for several ichthyoses. Indeed, many of the ichthyoses result from the mutation of genes encoding enzymes or cofactors involved in the metabolism of stratum corneum lipids.

#### Preclinical studies

TG1 deficient skin in ARCI is a good candidate for ERT [83]. Aufenvenne et al*.* [82] tested this in vivo with a mouse-model with humanized skin. They prepared liposomes with encapsulated recombinant human TG1. In-situ TG1 activity assays in cryosections showed a restoration of the TG1 activity. TEWL measurements of TG1-treated mice were compared to mice treated with retinoid cream. The TG1 group showed no increase in TEWL compared to normal human skin and indicated a restoration of the epidermal barrier, whereas the retinoid group presented a markedly increased TEWL value.

More recently, Plank et al*.* [84] used full-thickness skin equivalents derived from fibroblasts and keratinocytes of *TGM1*-related ARCI patients. They applied TG1 topically using thermoresponsive nanogels. Improvement of barrier function was tested with Lucifer yellow permeability tests. Before treatment, a 59-fold increase of the amount of Lucifer yellow passing through patient skin equivalents was found, compared to normal keratinocytes. After treatment this was decreased to 1.2. This indicated signs of epidermal barrier function restoration of the TG1-deficient skin in vitro.

Protein replacement therapy has also been studied for PSS1. Valentin et al*.* [36] conducted an in vitro study with corneodesmosin-deficient human epidermal equivalents. A specific carrier system was developed for delivery of liposome encapsulated recombinant human corneodesmosin. Immunofluorescence showed corneodesmosin staining at the transition of stratum corneum to stratum granulosum. However, the staining of healthy control epidermal equivalents was stronger and more pericellular. Results also showed alterations of the stratum corneum after treatment, comparable to normal equivalents. Furthermore, improved barrier function was achieved, as studied by toluidine blue penetration assay.

Grond et al*.* [85] investigated in vivo mouse-models with ARCI based on a *PNPLA1* deficiency. They found that topical application of epidermal lipids from wild type mice gave a 14-fold increased level of covalent linkage of ω-O-acylceramide to corneocyte proteins in newborn *PNPLA1*-deficient mice, then in mutant skins treated with epidermal lipids from *PNPLA1*-deficient mice. Mauldin et al*.* [86] performed an in vivo study, where they topically applied ω-O-acylceramides in *NIPAL4*-mutated dogs. As shown by electron microscopy analysis of skin biopsies from affected dogs, the treatment normalized the cornified lipid envelope. However, it failed to correct the clinical phenotype in affected canines. This was probably due to cytotoxicity attributable to accumulation of free fatty acids or other proximal metabolites.

#### Clinical studies

Lipid replacement could be effective for CHILD syndrome, which is caused by a deficiency in bulk cholesterol. In 2011, Paller et al*.* [87] proposed topical application of lovastatin and cholesterol. They reported two CHILD affected individuals, who displayed ichthyosiform plaques. They were treated twice daily with a 2% lovastatin/2% cholesterol lotion. After 6 months of treatment, treated areas were almost completely normalized. More recently, Bergqvist et al*.* [42] presented two cases of CHILD syndrome. One 11-year-old girl with a verruciform xanthoma was treated twice a day with 2% cholesterol and 2% lovastatin cream. After 4 weeks, the lesion had cleared. After 8 months, a recurrence of the lesion was successfully treated and the lesion was still in remission after 7 months being off treatment. However, the cream had no effect on a verruciform xanthoma on the foot of the second patient. Addition of 12% glycolic acid once a day resulted in a substantially decrease of the xanthoma after 12 months. Similar results were reached in another case report about a 2-month-old girl with lesions on the legs, also treated with 2% cholesterol and 2% lovastatin lotion twice a day [88]. Additionally, several case reports have shown promising results of simvastatin monotherapy for CHILD syndrome associated skin lesions. Yu et al*.* [89] reported four patients who were treated with simvastatin lotion. First, 2.5% simvastatin lotion was used, and after 3 months the individuals received 5% simvastatin. Skin lesions slightly improved after treatment with 2.5% simvastatin, but a marked improvement was seen after switching to 5% simvastatin. Other case reports of simvastatin monotherapy show similar responses [90, 91].

In conclusion, enzyme and lipid replacement are very interesting strategies to improve ichthyosis symptoms, by directly substituting the deficient substance. More studies should be encouraged to look at the clinical effect and consider the possible toxicity arising from accumulation of metabolic intermediates. There are currently no registered ongoing clinical trials.

### Gene therapy

Gene therapy is aiming to restore the wild type gene function. In another genodermatosis, epidermolysis bullosa (EB), gene therapy has led to promising results. Junctional EB patients have been successfully transplanted with genetically corrected autologous skin grafts [92, 93]. Gene therapy can be achieved by different approaches. A classical approach is introduction of the wild type gene copy into cells, using a viral vector. However, there are safety concerns regarding insertional mutagenesis that comes with the use of a viral vector [94, 95]. Also, introduction of viruses leads to production of antibodies, which could affect subsequent transduction of the same virus [96].

Another technique is gene editing. With gene editing, sequence specific nucleases are used to induct artificial double strand DNA breaks. This will activate the natural DNA repair system, without the risk of random integration of the transgene as with the use of a viral vector. Examples of nucleases are transcription activator-like effector nuclease (TALEN) and the bacterial CRISPR/Cas9 nuclease system [97]. Base editing is one of the most recent techniques of gene editing. With base editing, single nucleotide variants are introduced into DNA or RNA in living cells. It directly modifies nucleobases by introduction of point mutations, without DNA cleavage. Two forms of DNA base editors are adenine base editors (ABE) and cytosine base editors [98]. Furthermore, one could use small interfering RNA (siRNA) to silence mutated alleles in dominant disorders.

#### Preclinical studies

In 1997, Freiberg et al. [99] have explored gene therapy for XLI. They transduced the *STS* gene with a retroviral vector to primary keratinocytes derived from XLI patients. In vitro restoration of STS protein expression and STS enzymatic activity was seen*.* Transduced XLI keratinocytes were then grafted onto immunodeficient mice. After grafting, the transduced XLI epidermis showed STS expression by immunostaining, and a normalization of TEWL and histologic appearance. No further research regarding gene therapy for XLI has been found after this study.

Akayima et al. [20] have proposed the possibility of gene therapy for HI. They have cultured keratinocytes from HI patients and performed corrective gene transfer of the *ABCA12* gene with a pCMV-tag4B vector. Before genetic correction, double immunostaining showed a congested glucosylceramide distribution pattern in HI. Glucosylceramide is a major lipid component of the lamellar granules and is essential for the epidermal permeability barrier. After corrective gene transfer, a significant increase in number of cells that displayed a normal glucosylceramide distribution pattern was seen (from 6.98 to 16.7%), indicating recovery of lamellar granule lipid secretion.

In 2005 and 2006, Haug et al*.* [33, 100] have already provided promising results in vitro for gene therapy in SLS. They first conducted a study where they transferred FALDH in keratinocytes using recombinant adeno-associated virus-2 vectors (rAAV-2) in a hamster model. This resulted in a normal FALDH expression. One year later they applied the same technique on keratinocytes from human SLS patients, where they achieved an increase of 60–70% from normal FALDH activity.

March et al*.* [101] used TALENs for gene editing of the *KRT10* gene in EI. In this ex vivo study, they targeted a specific region of the *KRT10* gene known to cause a premature stop, leading to a genetic knockout. They tested the TALEN on keratinocytes derived from EI patients with mutant *KRT10* alleles, using modified single cell clones and murine xenodraft models. The on-target activity was measured and demonstrated strong gene disruption. Off-target activity was not observed. They suggested that epidermal generation from a single-cell clonal expansion from gene-edited keratinocytes is feasible.

For NS, Krystal Biotech inc. developed a replication-defective herpes simplex virus type 1 (HSV-1) vector encoding human *SPINK5* (KB104) for topical administration. HSV-1 can penetrate the skin and does not insert itself into the genome of the host. They presented their results at the annual meeting of the Society for Investigative Dermatology (2019). In the abstract provided of this in vitro study, human keratinocytes that were administered KB104 produced functional SPINK5 and were able to inhibit KLK5. Real time PCR and immunohistochemistry revealed that topical or intradermal administration of KB104 in mice resulted in expression of human *SPINK5*.

Regarding LI, Choate et al. [102] have investigated potential effects of gene therapy in vitro. They have used a retroviral vector to transfer the *TGM1* gene into keratinocytes of LI patients. In > 98% of the cells, expression of functional TG1 protein was achieved, resulting in restoration of epidermal architecture and barrier function.

The potential of gene therapy for *TGM1* deficiency has further been explored by Freedman et al. [103] The same strategy as described above for KB104 was used for TG1-deficient ARCI patients, where HSV-1 encoded for human TG1 (KB105). KB105 was analyzed in vitro with patient derived keratinocytes and in vivo, using murine models. This resulted in increased TG1 protein expression, whereas the control group showed no TG1 expression. Assessment of toxicity showed no adverse effects. Investigation of topical gene therapy has also been done by Gurevich et al*.* [104] for recessive dystrophic epidermolysis bullosa (RDEB), which is caused by mutations in the *COL7A1* gene that normally encodes for collagen VII (C7). They investigated beremagene geperpavec (B-VEC), a replication defective HSV-1 vector containing two copies of the *COL7A1* coding sequence*.* First, they evaluated the effect of B-VEC on C7 expression in keratinocytes and fibroblasts cultured of RDEB patients in vitro*.* Results showed restored C7 expression after 48 h of treatment. They administered intradermal B-VEC injections to C7-deficient mice in vivo, and observed C7 expression at day 3 and 7 by indirect immunofluorescence microscopy, and also measured *COL7A1* DNA and C7 transcript expression levels. This was further confirmed in human RDEB skin xenografted onto immunodeficient mice, treated with placebo or B-VEC injections. Control grafts showed dermal-epidermal separation, which was not observed in the xenografts treated with B-VEC. Immunofluorescence showed C7 expression in the xenografts after B-VEC, and was absent in the control grafts.

Dang et al. [105] have investigated the potential of base editing with the ABE system in human embryos. They first generated a homozygous mutant cell model with a c.607C > T mutation in the *TGM1* gene. Two different ABEs, called ABEmax-NG and Sc-ABEmax, showed potential in correcting the pathogenic mutation. Subsequently, oocytes and sperm of a couple both carrying a heterozygous c.607C > T *TGM1* mutation were used to create heterozygote mutant human embryos by in vitro maturation and intracytoplasmic sperm injection. In the ABEmax-NG group, seven embryos were collected and two of them displayed a completely wild genotype. In the Sc-ABEmax group, five of the eight embryos showed a completely wild genotype. The normalized editing efficiency for the ABE systems was 73.8% and 78.9%, respectively. DNA off-target analysis was performed by whole-genome sequencing and deep sequencing and showed no obvious off-target activity. However, a higher number of RNA single nucleotide variations was seen in embryos injected with the ABE system. Improvement of editors could reduce these RNA off-target effects and thus improve safety.

The technique of siRNA has been explored by Lee et al*.* [106] for KID syndrome*,* applied on keratinocytes with mutations in the *GJB2* gene. This in vitro study demonstrated inhibition of the mutated *GJB2* allele in the keratinocytes, indicated by improvement of its gap junction and hemichannel function. This could potentially improve the skin changes. A human-murine chimeric skin graft model was developed, and a way for a topical approach of siRNA on this in vivo model is currently under investigation.

#### Clinical studies

To further explore the potential of the above described KB105, Krystal Biotech Inc. presented interim results of a phase 1/2 placebo-controlled trial in *TGM1* associated ARCI patients at the annual meeting of the Society for Investigative Dermatology (2021). Three adults were included and received either topical KB105 or placebo. Biopsies were taken from KB105-treated areas to evaluate the effects. Treated areas showed detectable functionally active TG1, confirmed by quantitative real-time PCR, immunofluorescence and in situ analysis. Clinically, the treated areas showed a reduction of two points on the Investigator’s Global Assessment (IGA) scale.

Di et al*.* [107] generated gene-modified epithelial sheets using keratinocytes of NS patients and transduced them with a lentiviral vector encoding SPINK5. Successful generation of epithelial sheets was achieved in three of the six included patients, and one subject was suited for engraftment. After 1-month, complete healing had occurred with mild hypopigmentation and KLK5 expression similar to that in normal skin. However, KLK5 expression decreased again by month 3, 6 and 12. The treatment was considered safe and feasible.

The results of Gurevich et al*.* [104] regarding B-VEC for RDEB patients as described above, were further investigated in a phase I/II trial. In this trial, nine RDEB patients received B-VEC treatment. Wound area surface was treated topically with B-VEC or placebo for 12 weeks. B-VEC treated wounds were statistically significant different (*P* = 0.0026) from placebo treated wounds, based on wound closure responder analysis. However, results showed a numerically favorable trend towards B-VEC regarding time to and duration of wound closure. At weeks 8, 10 and 12, a statistically significant effect (*P* < 0.025) of reduction in wound surface area was seen. There were no serious or significant adverse events observed. This treatment focusses however on wounds and blisters, and not on the intact epidermis, which is different than in case of ichthyosis patients. One could thus wonder if the penetration and delivery with an HSV vector in ichthyosis would have the same efficacy as it has in EB.

#### Natural gene therapy

Revertant mosaicism is a phenomenon in which cells containing mutated copies of the gene undergo a second genetic event that leads to spontaneous correction of the gene [108]. This manifests itself on the skin as areas that seem phenotypically normal. This mechanism has been described in all forms of EB. Attempts to use revertant mosaicism as a therapy have not yet been very successful. However, one case concerning a junctional EB patient reports punch grafting of the revertant spot, with transplantation of the biopsy specimen. This resulted in re-epithelization of the wound with wild type skin [109]. One ichthyosis subtype, CRIE, displays the same phenomenon, but no studies have been published about the usage of reverted keratinocytes as therapy for CRIE [95, 108].

Gene therapy has not yet been explored as widely as biologicals have. The few studies that have been published show that gene therapy could be a safe and effective option, but further investigation is essential. Ongoing, registered clinical trials (Fig. 1) include the above described KB105 for *TGM1*-related ARCI, and a study about grafting of autologous epidermal sheets generated from genetically modified skin stem cells with a lentiviral vector for NS.

## Conclusion and future

In the past few years, relevant research regarding the molecular pathophysiology of ichthyosis has been conducted. This knowledge provides a foundation to target defective genes or the proteins that they encode for. These approaches include small molecules, protein and lipid replacement, and gene replacement and editing therapy. To date, these approaches are still in (pre-)clinical stage, ranging from phase I to III for clinical research. There is a need for more safety data, before these treatment options can be considered for application in humans. Only a few gene therapies are currently considered in the treatment of ichthyosis. Several therapies, such as the use of siRNA and CRISPR/Cas9, are already in the pre-clinical testing for EB, where they show promising results. These therapies have not yet been explored for ichthyosis, but could lead to new perspectives in the search for a cure. Next to the development of new therapies, drugs used for other diseases have been repurposed as well. Due to increasing knowledge about the immune profile, usage of biological therapeutics was considered in the treatment of ichthyosis. These biologics are already widely prescribed for other indications and are considered relatively safe. A growing amount of reports is available on this topic. Nonetheless, the inflammatory dysregulation in ichthyotic skin has only been explored in a few ichthyosis forms and with a relatively small patient number. Further research should investigate biomarkers in larger cohorts including all ichthyosis variants. Studies with long-term follow-up are necessary to show if the effects of biologics in ichthyosis are durable. Different treatment strategies, acting on different levels of the underlying pathophysiology, might be combined in one patient to optimize the clinical results. Furthermore, the applicability of pathogenesis-based therapies should be addressed, since they are likely to be gene-, or even individual-specific.

## Data Availability

Data sharing not applicable to this article as no datasets were generated or analysed during the current study.

## Abbreviations

ABE: Adenine base editor
AD: Atopic dermatitis
ARCI: Autosomal recessive congenital ichthyosis
B-VEC: Beremagene geperpavec
CAPE: *CARD14* associated papulosquamous eruption
CIE: Congenital ichthyosiform erythroderma
CHILD syndrome: Congenital hemidysplasia with ichthyosiform erythroderma and limb defects syndrome
CRIE: Congenital reticular ichthyosiform erythroderma
C7: Collagen VII
DSG1: Desmoglein-1
DSP: Desmoplakin
EI: Epidermolytic ichthyosis
ELA2: Epidermal elastase 2
ERT: Enzyme replacement therapy
FALDH: Fatty aldehyde dehydrogenase
HI: Harlequin ichthyosis
IASI: Ichthyosis assessment severity index
IASI-E: Ichthyosis assessment severity index-erythema
IASI-S: Ichthyosis assessment severity index-scaling
IGA: Investigator’s Global Assessment
IL: Interleukin
JAK: Janus kinase
K_i_: Inhibitory constant
KID syndrome: Keratitis–ichthyosis–deafness syndrome
KLK: Kallikrein-related peptidase
KRT1: Keratin-1
KRT10: Keratin-10
LEKTI: Lympho‐epithelial Kazal‐type‐related inhibitor
LI: Lamellar ichthyosis
NS: Netherton syndrome
ILC: Ichthyosis linearis circumflexa
SE: Scaly erythroderma
NRS: Numeric rating scale
PAR-2: Protease-activated receptor 2
PSS1: Peeling skin syndrome type 1
Raav-2: Recombinant adeno-associated virus-2
RDEB: Recessive dystrophic epidermolysis bullosa
SAM: Severe dermatitis, multiple allergies and metabolic wasting
siRNA: Small interfering RNA
SLS: Sjögren–Larsson syndrome
SFTI: Sunflower trypsin inhibitor
STS: Steroid sulfatase
TALEN: Transcription activator-like effector nuclease
TEWL: Transepidermal water loss
TGM1: Transglutaminase-1 (gene)
TG1: Transglutaminase-1 (protein)
tNG: Thermoresponsive nanogels
Th: T-helper cell
TNF-α: Tumor necrosis factor alpha
VIIS: Visual Index for Ichthyosis Severity
XLI: X-linked recessive ichthyosis
